# Education Research: The Effect of an X + Y Schedule Model on Neurology Residency Training

**DOI:** 10.1212/NE9.0000000000200017

**Published:** 2022-11-18

**Authors:** Shuvro Roy, Katherine Fu, Timothy E. Ryan, Yvette Bordelon, Charles C. Flippen, Adrienne M. Keener

**Affiliations:** From the Department of Neurology (S.R.), Johns Hopkins University, Baltimore, MD; Department of Neurology (S.R., K.F., T.E.R., Y.B., C.C.F., A.M.K.), UCLA, Los Angeles, CA; and Department of Neurology (T.E.R.), Cedars Sinai Medical Center, Los Angeles, CA.

## Abstract

**Background and Objectives:**

There is a need for earlier outpatient exposure in neurology training. In 2017, 56% of residents on the American Academy of Neurology (AAN) Graduating Resident Survey reported that they felt that the fellowship process started too early, and 46% felt that they did not have adequate outpatient exposure before making a fellowship decision. In addition, the traditional front-loaded resident schedule may contribute to high rates of burnout due to greater work hours and heavier inpatient load, as was suggested in the findings of a 2016 AAN survey comparing burnout among residents and fellows.

**Methods:**

We created an X + Y model within the UCLA Neurology Residency Program in the 2020–2021 academic year with the goal of increasing outpatient exposure earlier in training. We used a preintervention/postintervention design assessing measures of resident satisfaction, outpatient clinic exposure, number of inpatient handoffs, resident work hours, and scores on the resident in-training examination (RITE). We hypothesized that outpatient clinic exposure would increase, handoffs would diminish, work hours would be reduced, measures of resident satisfaction with inpatient care, outpatient care, and well-being would improve, and that RITE scores would improve. Work hours, handoffs, and number of clinic days were compared across each year via analysis of the resident schedule. Resident perceptions were obtained via an online survey at the end of their PGY-2 year. RITE scores were compared across a variety of subspecialties.

**Results:**

In the postintervention year, handoffs were reduced by 6.13 (95% CI 4.73–7.54) per week. Average clinic half-days increased by 4.51 (95% CI 7.76–0.53). Resident responses regarding their outpatient experience improved from 42% to 93% satisfied and from 60% to 94% satisfied for their inpatient experience. There was no difference in average work hours per week before and after the intervention. Regarding resident well-being, responses improved from 42% satisfied in the traditional model to 96% in the X + Y model. Among the RITE subjects covering primarily outpatient subspecialties, scores improved in each category.

**Discussion:**

After implementation of an X + Y model, we observed an improvement in outpatient exposure, learning and career satisfaction, and resident education on subspecialty topics.

Neurology as a field is a rapidly evolving environment. Neurology graduate medical education has continued to adapt to match that dynamic.

Neurology residencies have long struggled with providing adequate outpatient training.^1^ A 1994 survey of American neurology residency program directors reported a consensus that “current approaches to teaching in the outpatient setting fall short of an educationally ideal system.” It was reported that residents were spending 23% of their time in outpatient clinics, an amount thought to be inadequate. As the burden of neurologic disease has increased, so has the need for greater subspecialty neurology training.^2^ Training continues to be heavily inpatient focused and front loaded, making it difficult for trainees to develop strong outpatient general neurology skills and choose a subspecialty focus.^3^

In 2017, 90% of residents on the American Academy of Neurology (AAN) Graduating Resident Survey reported that they would be pursuing a fellowship. However, 54% felt that the fellowship application process started too early, and 46% did not feel that they had adequate outpatient exposure before making a decision.^4^ Eight of the top 10 most commonly pursued fellowships were outpatient specialties. Currently, the majority of US neurology residencies place an emphasis on inpatient training during Post-Graduate Year 2 (PGY-2, first year of neurology training).^5^ This is notable given the ongoing accelerated timeline for fellowship applications. In the 2017 AAN Program Directors survey,^6^ 78% of the 106 program directors indicated that the residency fellowship application process starts too early, and many favored delaying the application process to late in the PGY-3 year to allow for greater subspecialty exposure and exploration of career options before committing.

Previous AAN research has identified a high rate of burnout among neurology trainees, with many of the associated factors tied to work schedule.^7^ Seventy-three percent of 212 neurology residents surveyed in 2016 described at least 1 symptom of burnout (high emotional exhaustion, high depersonalization, or low personal accomplishment), which was higher than the burnout rate of fellows also surveyed (55%). Among the key differences between residents and fellows, residents spent 10 times as much time per year on inpatient coverage, spent more weekends in the hospital per year (23:4), and cared for fewer outpatients per week (6:10). On pooled analysis of all residents surveyed, the characteristics most closely associated with a lower burnout risk were work-life balance and meaningful work. In addition, trainees who reported their work meaningful were more likely to be satisfied with their career choice. Transitioning to a more outpatient-weighted schedule could help address several of these underlying stressors.

Given this landscape of neurology training, a new X + Y schedule was created at the University of California, Los Angeles (UCLA) Neurology Residency Program in lieu of the traditional neurology residency schedule during the PGY-2 year. We compared the number of inpatient handoffs, resident work hours, total number of continuity clinics, resident satisfaction of inpatient and outpatient care, markers of well-being, fellowship decision-making, and resident in-training examination (RITE) scores before and after introduction of the X + Y scheduling. Our hypothesis was that the transition to this new schedule would lead to a reduced number of handoffs, a reduction in resident work hours, an increased number of continuity clinics, increased satisfaction with outpatient care, improved resident well-being, enhanced fellowship decision-making, and improvement in RITE scores, particularly on questions related to outpatient subspecialties.

## Methods

### Planning the Intervention

**Figure 1:**
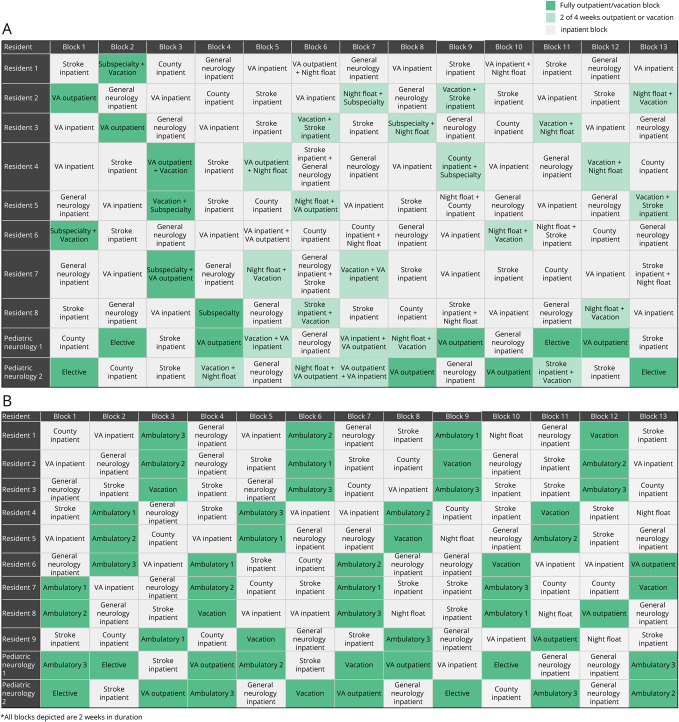
Preintervention and Postintervention Block Schedules (A) Sample traditional model block schedule. Each block represents a 4-week rotation, and this figure demonstrates the entire year's schedule. Fully ambulatory blocks are highlighted in dark green, and inpatient blocks are highlighted in gray. In some instances, residents were in 2-week rotations to accommodate vacation, or a 2-week night float rotation, and these blocks are highlighted in light green. For the Pediatric Neurology residents, there were additional VA outpatient rotations to meet ACGME requirements for outpatient exposure. (B) Sample X + Y block schedule. Each block represents a 2-week rotation, and this sample represents only half of the year for readability. Ambulatory blocks are highlighted in green, and inpatient blocks are highlighted in gray. Vacations were substituted in place of ambulatory blocks, and Pediatric Neurology rotations were scheduled in accordance with their specific ACGME requirements. Starting in block 8, the PGY-2 residents start covering night float, and so with that one fewer PGY-2 was present on the VA inpatient team. The design of the schedule was such that at least for every 4 weeks on an inpatient service, residents would either have an ambulatory block or vacation for 2 weeks. In some cases, residents may have had an ambulatory block after 2 weeks on an inpatient service for the sake of balancing the schedule. Each ambulatory block was associated with different subspecialty clinics. PGY-2 = Post-Graduate Year 2; VA = Veterans Affairs.

We used a preintervention/postintervention design in assessing outcomes. In the 2020–2021 academic year, the UCLA Neurology Residency Program adopted a 4 + 2 schedule for the PGY-2 class (11 residents in total, 9 adult neurology residents and 2 pediatric neurology fellows completing their adult neurology training year), with 2 weeks of outpatient rotations for every 4 weeks on inpatient rotations. This specific variation of X + Y was used to create appropriately sized resident cohorts for the PGY-2 class size and different sites requiring coverage. We created schedule templates using variably spaced 2-week inpatient neurology blocks at 3 different hospital sites interspersed with 2-week outpatient clinic blocks. The resident continuity clinics in both models were split between the main academic hospital and the Veterans Affairs (VA) outpatient center. In the traditional model, PGY-2 residents primarily covered inpatient teams at the VA, County, or the main university hospital with limited dedicated outpatient time, and rotations were 4 weeks long. When designing the master template, we used a modified version of the process outlined by Internal Medicine residency programs^8^ (Figure 1). Necessary modifications were made to accommodate the Accreditation Council for Graduate Medical Education (ACGME) requirements for pediatric neurology residents completing their adult neurology training year,^9^ in which residents are required to have 6 months of inpatient rotations and 3 months of both outpatient adult neurology and elective.

### Intervention

**Figure 2:**
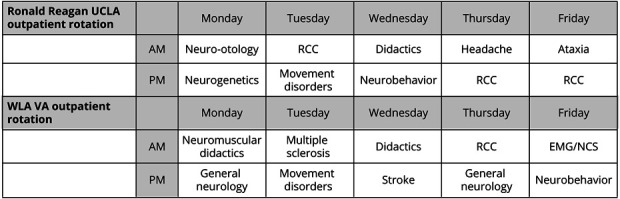
Sample Ambulatory Block Sample clinic schedules during their +2 outpatient block illustrating the variety of subspecialty clinics neurology residents rotate through in 1 week. Sample schedules are provided for the Ronald Reagan UCLA outpatient site and the WLA VA outpatient site. RCC = resident continuity clinic; VA = Veterans Affairs; WLA = West Los Angeles.

In both the traditional model and the 4 + 2 schedule, continuity clinics were scheduled during the morning and afternoon sessions, with an average of 3.5 patients seen per clinic. In the traditional model, residents rotated through 4-week rotations and had a weekly half-day continuity clinic, regardless of whether they were on an inpatient or outpatient rotation (Figure 1A). However, in the 4 + 2 schedule, all continuity clinics were removed from inpatient rotations for the PGY-2 class and scheduled within the +2 clinic weeks (Figure 1B). In both years, residents each had their own cohort of clinic patients. The PGY-2 residents all continued to meet the ACGME requirements of 40 continuity clinics/y, not separated by greater than 5 weeks (with the allowed exception of vacation). Finally, the clinic blocks had additional subspecialty clinics built into the mornings and afternoons during which residents were not in their continuity clinics. These subspecialty clinics included Ataxia, EMG/NCS, Headache, Movement Disorders, Multiple Sclerosis, Neurobehavior, Neurogenetics, Neuromuscular, Neuro-oncology, Neuro-otology, Sleep, and Stroke (Figure 2).

### Variables

We had two primary outcomes. The first was the validated Learners’ Perception Survey, given to both pre-intervention and post-intervention classes, to assess attitudes towards inpatient care, outpatient care, and overall well-being. The second was outpatient exposure as counted by the number of resident continuity clinics and total weeks on an outpatient rotation. Additional secondary outcomes in both classes were RITE scores, number of inpatient handoffs, and work hours.

### Data Sources/Measurement

We surveyed the 2019–2020 PGY-2 resident physicians (preintervention class) in April of the year before implementation of the 4 + 2 schedule and the 2020–2021 PGY-2 resident physicians (postintervention class) who participated in the new schedule the following April. We used a retrospective pre-post survey design and sent a series of questions from the validated Learners' Perception Survey via an anonymous Qualtrics software link.^10^ Survey completion was optional, and residents did not receive any compensation for completion. Most questions were in a 5-point Likert scale format querying satisfaction with statements on outpatient and inpatient experience. The survey was divided into 3 sections: outpatient experience, inpatient experience, and readiness for fellowship. Specific questions were marked for a subgroup analysis assessing trainee well-being based on factors significantly associated with burnout and career satisfaction in the 2016 AAN survey (meaningful work and work-life balance). We added questions on the residents' satisfaction in making a fellowship decision. Because of the small sample of a neurology residency class, we analyzed these survey results in a descriptive manner. “Neither satisfied nor dissatisfied” was reported as a neutral response. “Extremely satisfied” and “Somewhat satisfied” were reported as satisfied responses. “Somewhat dissatisfied” and “Extremely dissatisfied” were reported as unsatisfied responses. No programmatic changes beyond the X + Y schedule were made to the residency that were expected to alter responses.

The number of resident clinics and the total outpatient time before and after implementation of the X + Y schedule were tabulated via analysis of the online schedule. To assess the effect of the schedule on resident work hours, we sampled the weekly timesheets submitted by the neurology residents both preintervention and postintervention for each academic year.

We examined inpatient handoffs (number of instances in which a group of patients was handed off from one resident to another) across all classes before and after implementation of the X + Y schedule via analysis of the online schedule. At the main academic hospital, there were 3 handoffs that occurred each day: morning, long call, and overnight. At the VA and County sites, there were 2 handoffs that occurred on weekdays: morning and overnight. On weekends, there was one handoff. Any outpatient clinic scheduled for residents while rotating on an inpatient service and any time a resident rotated off service was counted as an additional handoff.

The 2020 and 2021 RITEs were administered in February of each year to the corresponding PGY-2 classes. The 2020 examination consisted of 396 scored questions, and the 2021 examination consisted of 400 scored questions, each covering 8 different sections: “Neurocognitive Disorders and Movement Disorders,” “Vascular Neurology and Neurocritical Care,” “Neuromuscular,” “Epilepsy and Sleep Disorders,” “Adult Neuroimmunologic and Neuroinfectious Disease,” “Headache, Neuro-oncology, Neuro-ophthalmology, and Neuro-otology,” “Psychiatric Disorders,” and “Pediatric Neurology.” We used the yearly program result reports to compare performance in each year by subspecialty based on the additional rotations introduced through the X + Y model. Notably, within the design of the UCLA Neurology Residency Program, Pediatric Neurology and Psychiatry rotations occur in the PGY-3 and PGY-4 years, respectively. Thus, we combined the weighted scores for these sections to serve as a comparison for the other sections above as neither class had exposure to these rotations at the time of taking the RITE. We did so by adding together the correct and total number of questions in each section (190 in 2020 and 143 in 2021 for Psychiatry; 720 in 2020 and 924 in 2021 for Pediatric Neurology) and calculating a combined score for each class. Exposure to inpatient Vascular Neurology and inpatient Epilepsy was unchanged across each class outside the overall reduction in inpatient rotations. The majority of EEG education occurs during the PGY-3 year, and PGY-2s are expected to contact fellows or attendings for EEG interpretation.

### Statistical Methods

The variables of number of continuity clinics, handoffs, and work hours were analyzed by the unpaired *t* test. We defined statistical significance as *p* < 0.05.

RITE results were tested for significance via the 2-tailed Z-test for comparison of proportions with the assumption of a normal distribution nationally for the RITE examination. After correction for multiple comparisons across 7 subspecialty areas via Bonferroni correction, statistical significance was defined as *p* < 0.007. All statistics were computed using Microsoft Excel (Redmond, WA). The study was reviewed and granted exempt status by the UCLA Institutional Review Board, and deidentified data were provided to the authors for analysis.

### Data Availability

The data sets generated during and/or analyzed during the current study are not publicly available to ensure the confidentiality of the participating residents, but anonymized data are available from the corresponding author on reasonable request.

## Results

### Participants

There were 10 residents in the preintervention class (8 adult neurology and 2 pediatric neurology) and 11 residents (9 adult neurology and 2 pediatric neurology) in the postintervention class. In the preintervention class, 8 residents were female and 2 were male, and the average age was 32.6 (±2.37) years. In the postintervention class, 7 residents were male and 4 were female, and the average age was 30.4 (±2.18) years.

### Inpatient vs Outpatient Time

In the traditional model class, the average number of weeks on inpatient rotations was 44.1 (±0.94) and 3.9 (±0.94) weeks on outpatient rotations. In the X + Y class, the residents averaged 32.7 (±3.84) weeks on inpatient service and 15.3 (±3.84) weeks on outpatient rotations.

### Continuity Clinics

Residents in the preintervention year averaged 39.3 clinic half-days per year and 43.5 clinic half-days per year in the postintervention year, with a mean difference of 4.51 (95% CI 7.76, 0.53, *p* = 0.026). Across all residents, this totaled an additional 154 patient appointment slots added to the postintervention year. There was no difference in the no-show rate between classes.

### Handoffs

In examining the effect of the 4 + 2 schedule on inpatient handoffs, we found that there was an overall reduction. Before the X + Y implementation, there were 1,874 inpatient handoffs for the preintervention year, an average of 36.7 per week across all 3 inpatient sites. After X + Y implementation, there were 1,561 inpatient handoffs in the postintervention year, an average of 30.6 per week across all 3 sites. Between the 2 years, there was a reduction of 6.13 handoffs per week (95% CI 4.73–7.54, *p* < 0.0001). However, there was an increase in end-of-rotation handoffs, from 143 to 188, due to 2-week rotations, instead of 4 weeks.

### Learners' Perceptions

**Figure 3:**
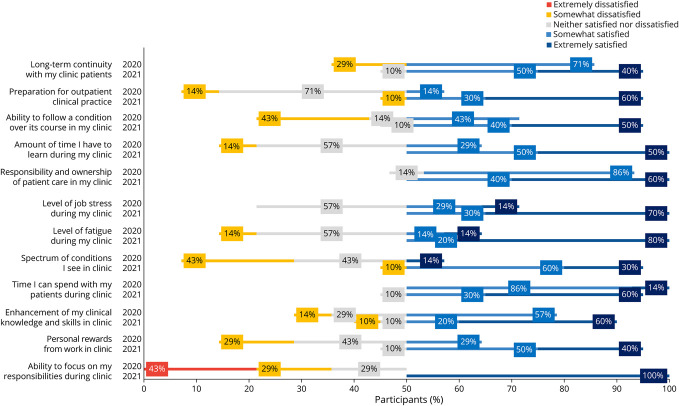
Outpatient Learner Perception Survey Data

**Figure 4:**
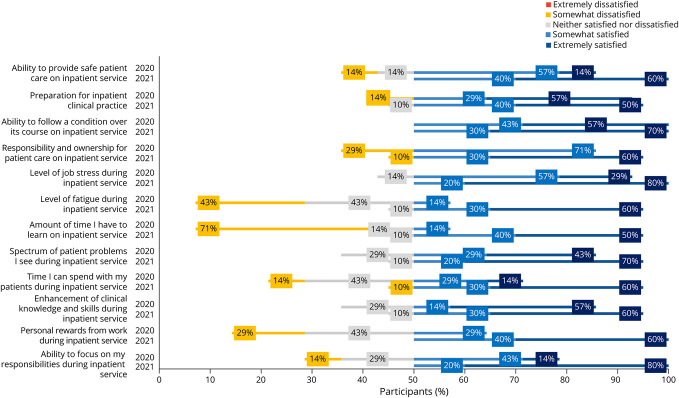
Inpatient Learners' Perception Survey Data

In all, 17 of 19 residents completed the survey (89% response rate). Seven of 17 respondents completed their PGY-2 year in the 2019–2020 academic year, and 10 of 17 respondents completed their PGY-2 year in the 2020–2021 academic year. Across all 13 outpatient questions, there was a greater percentage of satisfied responses in the postintervention class for each question compared with the preintervention class (Figure 3). Of 120 resident responses regarding outpatient experience in the postintervention class, 112/120 (93%) were satisfied, compared with 35/84 (42%) satisfied responses from the preintervention class. Of the 120 resident responses regarding inpatient experience, 113/120 (94%) were satisfied, compared with 50/84 (60%) satisfied responses from the preintervention class (Figure 4).

Notable improvements in resident satisfaction included preparation for outpatient practice (10% satisfied to 90% satisfied), level of job stress during my continuity clinic (42% satisfied responses to 100% satisfied responses), level of job stress during inpatient service (14% satisfied responses to 90% satisfied responses), level of fatigue during inpatient service (14% satisfied responses to 90% satisfied responses), personal reward from work during inpatient service (29% satisfied responses to 100% satisfied responses), and ability to focus on my outpatient responsibilities during continuity clinic (0% satisfied responses to 100% satisfied responses).

**Figure 5:**
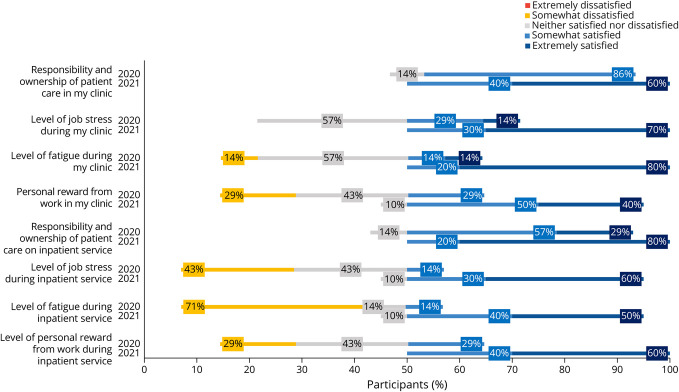
Trainee Well-Being Learners' Perception Survey Data

Eight questions focused on assessing resident burnout and well-being. Of 80 resident responses regarding well-being in the 2020–2021 class, 77/80 (96%) responses were satisfied, compared with 23/56 (41%) in the 2019–2020 class (Figure 5).

Six of 10 respondents in the 2020–2021 class reported satisfaction regarding “Being able to confidently make a fellowship decision,” compared with 0/10 respondents in the 2019–2020 class. For the postintervention PGY-2 class, 2 additional questions were added to gauge any effect of the 4 + 2 schedule on making a fellowship decision. Seven of 10 respondents stated that the “X + Y schedule was helpful in selecting a possible fellowship,” and 8/10 respondents stated that the “X + Y schedule was helpful in excluding a possible fellowship.”

### Work Hours

In each year, timesheets were pulled from each class from July to April to assess average hours worked per week across all residents. In the preintervention class, 289 total weekly timesheets were submitted out of a possible 351 (82% response rate). In the postintervention class, 314 weekly timesheets were submitted out of a possible 351 (89% response rate). The preintervention class averaged 61.3 ± 5.79 hours worked per week, and the postintervention class averaged 59.5 ± 3.23 hours worked per week. This difference was nonsignificant (*p* = 0.39).

### RITE Results

The results of the RITE for each class are detailed in Table. As described in the Methods, a composite score of Psychiatry and Pediatric Neurology was calculated for each class as a control. In the 2020 class, the resident average across these combined 91 questions (19 Psychiatry and 72 Pediatric Neurology) was 59.0%. In the 2021 class, the resident average across 97 questions (13 Psychiatry and 84 Pediatric Neurology) was 58.9%. In “Vascular Neurology and Neurocritical Care,” the 2020 class scored 66% (64th percentile), whereas the 2021 class scored 67% (74th percentile). Neither was a significant difference.

**Table:** Difference in Resident In-Training Examination Scores Across Subcategories

Topic	No. of questions (section)	Total sample	Percentage correct	% Difference	Percentile	*p* Value^a^
Neurocognitive Disorders and Movement Disorders						
Traditional model	77	770	61		51st	**0.006**
X + Y model	56	616	68	+7	87th	
Vascular Neurology and Neurocritical Care						
Traditional model	46	460	66		64th	0.743
X + Y model	45	495	67	+1	74th	
Neuromuscular^a^						
Traditional model	63	630	59		61st	0.045
X + Y model	40	440	65	+6	79th	
Epilepsy and Sleep Disorders						
Traditional model	39	390	67		14th	0.076
X + Y model	31	341	73	+6	77th	
Neuroimmunologic and Neuroinfectious Disease						
Traditional model	40	400	60		57th	0.122
X + Y model	46	506	65	+5	81st	
Headache and Pain Disorders, Neuro-oncology, Neuro-ophthalmology, and Neuro-otology						
Traditional model	39	390	64		58th	0.029
X + Y model	42	462	71	+7	87th	
Psychiatric Disorders + Pediatric Neurology						
Traditional model	91	910	59		n/a	1.0
X + Y model	97	1,067	59	+0	n/a	

a Two-tailed Z-test for comparison of proportions.

*p* < 0.007 for significance.

Among the subjects covering outpatient subspecialties, scores improved in each. “Neurocognitive Disorders and Movement Disorders” improved from 61% (51st percentile) to 68% (87th percentile) *p* = 0.029, “Neuromuscular” improved from 59% (61st percentile) to 65% (79th percentile) *p* = 0.045, “Headache, Neuro-oncology, Neuro-ophthalmology, and Neuro-otology” improved from 64% (58th percentile) to 71% (87th percentile). The difference in the latter was statistically significant when corrected for multiple comparisons (*p* = 0.006). The former 2 categories had a *p* value of <0.05, but statistical significance was not reached after correcting for multiple comparisons, which was defined as *p* < 0.007. “Epilepsy and Sleep Disorders” improved from 67% (14th percentile) to 73% (77th percentile), and “Neuroimmunologic Disease and Neuroinfectious Disease” improved from 60% (57th percentile) to 65% (81st percentile), although neither improvement was statistically significant (*p* = 0.08 and *p* = 0.12, respectively).

## Discussion

We examined the implementation of an X+Y schedule in a neurology residency program, and assessed its effect on work hours, resident well-being, and resident education. Although this sort of model has been previously implemented in internal medicine (IM), including the majority of primary care programs^11-14^ and pediatrics^15^; the smaller classes of neurology programs present a unique challenge with implementation. The generalizability of the X + Y model to individual neurology programs will depend on the rotation and coverage requirements, optimal team structure, ideal permutation of inpatient and ambulatory blocks, and learner pool. We executed an X + Y schedule with a class of 11 residents across 3 different inpatient sites, while maintaining the 40 continuity clinic ACGME requirement within the ambulatory blocks. This suggests that smaller residencies could allow for adequate workflow distribution for resident coverage, which has been a long-standing concern for X + Y implementation.^16^

We observed several changes after instituting the new model. There was a reduction in the number of inpatient handoffs per week. Higher numbers of handoffs have been associated with worse outcomes in patient care.^17,18^ These findings were similar to those found by Osborn et al.^15^ in the implementation of an X + Y model in a pediatric residency program. Although there was an increase in the overall number of end-of-rotation handoffs, this was mitigated by an effort to maintain continuity on the same rotation when possible and the removal of clinic requirements during inpatient rotations. We did not track the adverse events that occurred in the preintervention and postintervention period and could not track patient satisfaction scores by resident, but the residents noted an improvement in their ability to provide safe care on the inpatient services on the Learners' Perception Survey.

We examined the implementation of the X + Y model and its effects on improving resident fellowship selection experience. Our findings before implementation were consistent with the national data obtained in the AAN large graduate survey in 2017, with no residents in the preintervention class responding that they could confidently make a fellowship decision. Six of 10 residents in the postintervention class reported satisfaction with their ability to make a fellowship decision.

Little data exist to date on the effect of an X + Y model on resident education, although prior studies have shown perceived improvements in educational experience.^8,11,15,16^ We had posited that with greater exposure to a wide variety of subspecialty clinics, we would see a corresponding improvement in RITE scores related to each topic. The scores across each class in the subspecialties covered in the PGY-3 and PGY-4 years, Psychiatry and Pediatric Neurology, were nearly identical at 59.0% and 58.9%, respectively. The scores for Vascular Neurology and Neurocritical Care, primarily inpatient specialties, also remained nearly identical for the preintervention and postintervention classes, at 66% and 67%, respectively, possibly indicating that the quality of inpatient education was not compromised despite a reduction in inpatient weeks.

Across the 4 subjects focused on subspecialty clinics introduced in the X + Y schedule (“Neuromuscular,” “Epilepsy and Sleep Disorders,” “Neuroimmunologic Disease and Neuroinfectious Disease,” and “Headache, Neuro-oncology, Neuro-ophthalmology, and Neuro-otology”), the postintervention class demonstrated an improvement in raw percentage and percentile in each. The average improvement across these subjects was 6.0%. The improvement was not quite significant in “Epilepsy and Sleep Disorders,” as well as “Neuroimmunologic Disease and Neuroinfectious Disease,” despite an improvement in percentile. This could be due to a relative lack of outpatient exposure in these disciplines, as EEG and Epilepsy rotations are focused in the PGY-3 year in our program, while the Multiple Sclerosis clinic was only present on one half-day of one of the 4 different clinic schedules. Alternatively, both of these areas represent problems commonly evaluated on the inpatient service, which is supported by prior examinations of common inpatient neurologic disease.^19,20^ Because of the grouping of Epilepsy with Sleep Disorders (a clinic added to the subspecialty pool during +2 weeks), this section was not included in the control set. Although RITE examination scores are imperfect measures of outpatient knowledge, it was interesting to note that across almost every subspecialty the residents had greater exposure to, there was a statistically significant improvement in performance before correction for multiple comparisons, whereas there was no significant change across the specialties primarily experienced on the inpatient side or in the PGY-3 and PGY-4 years. These data certainly warrant further evaluation and replication to assess the true effect of this schedule on resident education.

We hypothesized that with more outpatient rotations, there would be a significant reduction in average work hours; however, our data did not support this hypothesis. Previous studies in IM demonstrated an improvement in career satisfaction from 66% to 80% after implementation of ACGME work-hour restrictions. Despite a lack of change in work hours, satisfaction improved after our intervention.

Resident well-being and the promotion of resident wellness is also key to an improved residency experience, with published calls to action highlighting the importance of this issue in graduate medical education.^21-23^ The 2015 Resident Well-Being Survey highlights the importance of resident wellness in the field of neurology, which was tied for third to last when residents were asked whether they would select medicine as a career again.^24^ The AAN has recognized the importance of emphasizing trainee well-being.^25^ In addition, other neurology residency programs have studied factors influencing well-being at their own residency programs.^26^ We observed improved perceptions among the residents in both outpatient and inpatient settings (Figure 5). Although we did not administer surveys to specifically assess for burnout or well-being in residents, the questions of fatigue, job stress, and personal reward from work may serve as surrogates for assessments of emotional exhaustion and personal accomplishment, which are assessed in scales of burnout such as the Maslach Burnout Inventory,^27^ and the factors identified by Levin et al.^7^ to be associated with burnout. Our findings are consistent with those of prior X + Y models, which found that a majority of residents reported an improvement in level of fatigue and job stress in both inpatient and outpatient settings, when minimizing conflicts between inpatient and outpatient responsibilities.^15,19^ Implementation of the X + Y scheduling model serves as an example of a structural change that residency programs can implement to promote wellness among residents.

Limitations of this study include a small sample size of a single preintervention and postintervention class at a single institution. As such, our findings may not be generalizable to other institutions. In addition, we did not have a 100% response rate among the residents, which could have led to selection bias. We did not send surveys to prior graduates of the program due to risk of recall bias, and unfortunately, we could not compare RITE scores before the 2019–2020 academic year due to variations in RITE score reporting. However, the responses of the preintervention class were consistent with the national data obtained by the AAN in 2017 that residents overall felt dissatisfaction with their outpatient experience in preparation for future clinical practice. In addition, 8/10 residents in the postintervention group reported that they had previously been exposed to an X + Y schedule in their IM internship training, which may have confounded their responses. Both classes completed their PGY-2 year in the setting of the COVID-19 pandemic, although neither class was redeployed to another specialty, and there were no clinic closures in either class. At the height of pandemic surges, care delivery in many clinics was transitioned to telemedicine. The preintervention class completed their PGY-2 year during the prevaccine period, and this may have contributed to a sense of worsened well-being. By contrast, the postintervention class had access to the first COVID-19 vaccines halfway through their PGY-2 year, although they also worked through the delta variant surge. Ultimately, we cannot fully account for how the COVID-19 pandemic contributed to resident experience, but these results must be considered in that context. Residents may have had more time in the X + Y model to prepare for the RITE examination, although neither class was aware of this metric being studied, and no change was made in either RITE preparation or in didactic material. We also could not account for differences between the classes in inherent attitudes or test-taking aptitude.

Given the focus of modern neurology on greater subspecialty training, more comprehensive examination of the X + Y model on resident education is needed. In the future, we hope to survey residency classes on their experience with the X + Y model to better understand the specific components that led to improved inpatient and outpatient trainee experiences. Future replication of sustained RITE score improvement and whether early exposure to particular outpatient-based subspecialties contributed to these improvements will also be necessary. Surveying trainees at other institutions, as the X + Y model becomes more widely implemented in neurology residency, will also improve the reliability of these findings. Future exploration of the elements of the X + Y model that promote neurology trainee well-being is also warranted. In addition, the RITE scores offer only a proxy of outpatient knowledge, and further evaluation with other metrics such as patient satisfaction, patient outcomes, case logs, or faculty evaluations may provide a clearer understanding of outpatient neurology competence and diversity of subspecialty exposure.

For neurology programs considering an X + Y schedule, it is important to involve all stakeholders, including department leadership, inpatient, and outpatient faculty at all sites, as the schedule will have significant effects on their rotations. Our recommendation to programs hoping to implement an X + Y schedule is to begin by determining the ideal permutation of inpatient and outpatient weeks, based on the number of resident cohorts available, as detailed by Shalaby et al. The most common X + Y permutations in IM were 4 + 1, 6 + 2, and 4 + 2. For smaller programs, the proportion of outpatient weeks may be lower to maintain the integrity of the inpatient coverage. After this is decided, implementing the schedule may necessitate changes in the number of residents on inpatient and outpatient services. For example, to implement the schedule in our model, one resident was removed from the VA inpatient team once our PGY-2 residents began covering night float. It is important to factor in the early input of chief residents to determine which iteration of X + Y works best for the program, and whether it can be instituted while meeting ACGME requirements. Importantly, programs should be aware of the different ACGME requirements for pediatric neurology residents completing an adult neurology year regarding requirements for outpatient, inpatient, and elective time. Programs can apply for certain waivers from specific ACGME rules if serving an educational purpose, but in our case, this was not necessary. Finally, ongoing feedback from residents and faculty will be paramount to a successful transition.

The advent of X + Y models represents a significant change from the design of neurology residency programs over the last century. Current challenges facing training programs include a lack of outpatient exposure in the PGY-2 year, an early fellowship application schedule, and burnout among trainees. Based on the initial results of our implementation of an X + Y model within a neurology residency program, there is promise that this training model achieves the goals of increasing outpatient exposure, improving learning and career satisfaction, reducing burnout, reducing inpatient handoffs, and improving resident education.

## Appendix Authors

**Table:**

Name	Location	Contribution
Shuvro Roy, MD	Department of Neurology, Johns Hopkins University, Baltimore, MD	Drafting/revision of the manuscript for content, including medical writing for content; major role in the acquisition of data; study concept or design; and analysis or interpretation of data
Katherine Fu, MD	Department of Neurology, UCLA, Los Angeles, CA	Drafting/revision of the manuscript for content, including medical writing for content, and major role in the acquisition of data
Timothy E. Ryan, MD	Department of Neurology, UCLA, Los Angeles, CA; Department of Neurology, Cedars Sinai Medical Center, Los Angeles, CA	Drafting/revision of the manuscript for content, including medical writing for content, and major role in the acquisition of data
Yvette Bordelon, MD, PhD	Department of Neurology, UCLA, Los Angeles, CA	Drafting/revision of the manuscript for content, including medical writing for content, and study concept or design
Charles C. Flippen II, MD	Department of Neurology, UCLA, Los Angeles, CA	Major role in the acquisition of data and study concept or design
Adrienne M Keener, MD	Department of Neurology, UCLA, Los Angeles, CA	Drafting/revision of the manuscript for content, including medical writing for content; major role in the acquisition of data; study concept or design; and analysis or interpretation of data

## Glossary

AAN: American Academy of Neurology
ACGME: Accreditation Council for Graduate Medical Education
IM: Internal Medicine
PGY-2: Post-Graduate Year 2
RITE: resident in-training examination
VA: Veterans Affairs

## References

[1] Gelb DJ. Teaching neurology residents in the outpatient setting. Arch Neurol. 1994;51(8):817-820. doi: 10.1001/archneur.1994.00540200097022. 8042931

[2] Sarva H, Patino GA, Rashid M, Owens JWM, Robbins MS, Sandrone S. The status of neurology fellowships in the United States: clinical needs, educational barriers, and future outlooks. BMC Med Educ. 2021;21(1):108. doi: 10.1186/s12909-021-02536-8. 33596875 PMC7891131

[3] Naley M, Elkind MSV. Outpatient training in neurology: history and future challenges. Neurology. 2006;66(1):E1-E6. doi: 10.1212/01.wnl.0000191319.07563.eb. 16401831

[4] Mahajan A, Cahill C, Scharf E, et al. Neurology residency training in 2017: a survey of preparation, perspectives, and plans. Neurology. 2019;92(2):76-83. doi: 10.1212/WNL.0000000000006739. 30518554

[5] Ances B. The more things change the more they stay the same: a case report of neurology residency experiences. J Neurol. 2012;259(7):1321-1325. doi: 10.1007/s00415-011-6347-8. 22186851 PMC3358425

[6] London ZN, Khan J, Cahill C, Schuyler E, Wold J, Southerland AM. 2017 Program Director Survey: feedback from your adult neurology residency leadership. Neurology. 2018;91(15):e1448-e1454. doi: 10.1212/WNL.0000000000006315. 30194246

[7] Levin KH, Shanafelt TD, Keran CM, et al. Burnout, career satisfaction, and well-being among US neurology residents and fellows in 2016. Neurology. 2017;89(5):492-501. doi: 10.1212/WNL.0000000000004135. 28667180

[8] Shalaby M, Yaich S, Donnelly J, Chippendale R, DeOliveira MC, Noronha C. X + Y scheduling models for internal medicine residency programs-a look back and a look forward. J Grad Med Educ. 2014;6(4):639-642. doi: 10.4300/JGME-D-14-00034.1. 26140111 PMC4477545

[9] ACGME. Pediatric Neurology Program Requirements; 2020. Accessed April 14, 2021. acgme.org/Portals/0/PFAssets/ProgramRequirements/185_ChildNeurology_2020.pdf?ver=2020-02-25-140857-647.

[10] Byrne JM, Chang BK, Gilman SC, et al. The learners' perceptions survey-primary care: assessing resident perceptions of internal medicine continuity clinics and patient-centered care. J Grad Med Educ. 2013;5(4):587-593. doi: 10.4300/JGME-D-12-00233.1. 24455006 PMC3886456

[11] Heist K, Guese M, Nikels M, Swigris R, Chacko K. Impact of 4 + 1 block scheduling on patient care continuity in resident clinic. J Gen Intern Med. 2014;29(8):1195-1199. doi: 10.1007/s11606-013-2750-4. 24408278 PMC4099454

[12] Warm EJ, Schauer DP, Diers T, et al. The ambulatory long-block: an Accreditation Council for Graduate Medical Education (ACGME) Educational Innovations Project (EIP). J Gen Intern Med. 2008;23(7):921-926. doi: 10.1007/s11606-008-0588-y. 18612718 PMC2517908

[13] Wieland ML, Halvorsen AJ, Chaudhry R, Reed DA, McDonald FS, Thomas KG. An evaluation of internal medicine residency continuity clinic redesign to a 50/50 outpatient-inpatient model. J Gen Intern Med. 2013;28(8):1014-1019. doi: 10.1007/s11606-012-2312-1. 23595923 PMC3710381

[14] O'Rourke P, Tseng E, Chacko K, Shalaby M, Cioletti A, Wright S. A national survey of internal medicine primary care residency program directors. J Gen Intern Med. 2019;34(7):1207-1212. doi: 10.1007/s11606-019-04984-x. 30963438 PMC6614222

[15] Osborn R, Bullis E, Fenick AM, Powers E, Banker S, Asnes A. X + Y scheduling in pediatric residency: continuity, handoffs, and trainee experience. Acad Pediatr. 2019;19(5):489-494. doi: 10.1016/j.acap.2019.05.001. 31077879

[16] Mariotti JL, Shalaby M, Fitzgibbons JP. The 4-1 schedule: a novel template for internal medicine residencies. J Grad Med Educ. 2010;2(4):541-547. doi: 10.4300/JGME-D-10-00044.1. 22132275 PMC3010937

[17] Starmer AJ, Sectish TC, Simon DW, et al. Rates of medical errors and preventable adverse events among hospitalized children following implementation of a resident handoff bundle. JAMA. 2013;310(21):2262-2270. doi: 10.1001/jama.2013.281961. 24302089

[18] Singh H, Thomas EJ, Petersen LA, Studdert DM. Medical errors involving trainees: a study of closed malpractice claims from 5 insurers. Arch Intern Med. 2007;167(19):2030-2036. doi: 10.1001/archinte.167.19.2030. 17954795

[19] Stepczynski J, Holt SR, Ellman MS, Tobin D, Doolittle BR. Factors affecting resident satisfaction in continuity clinic-a systematic review. J Gen Intern Med. 2018;33(8):1386-1393. doi: 10.1007/s11606-018-4469-8. 29736753 PMC6082200

[20] Chowdhury RN, Hasan ATMH, Ur Rahman Y, Khan SI, Hussain AR, Ahsan S. Pattern of neurological disease seen among patients admitted in tertiary care hospital. BMC Res Notes. 2014;7:202. doi: 10.1186/1756-0500-7-202. 24684800 PMC3977680

[21] Salles A, Liebert CA, Greco RS. Promoting balance in the lives of resident physicians: a call to action. JAMA Surg. 2015;150(7):607-608. doi: 10.1001/jamasurg.2015.0257. 25992632

[22] Daskivich TJ, Jardine DA, Tseng J, et al. Promotion of wellness and mental health awareness among physicians in training: perspective of a national, multispecialty panel of residents and fellows. J Grad Med Educ. 2015;7(1):143-147. doi: 10.4300/JGME-07-01-42. 26217450 PMC4507916

[23] Ripp JA, Privitera MR, West CP, et al. Well-being in graduate medical education: a call for action. Acad Med. 2017;92(7):914-917. doi: 10.1097/ACM.0000000000001735. 28471780

[24] ACGME. Resident Wellness Data: Results from Five Years of Surveys; 2019. Accessed June 15, 2021. cpb-us-e1.wpmucdn.com/sites.uw.edu/dist/9/4123/files/2019/12/SES_092.original.1552146317.pdf.

[25] American Academy of Neurology. Wellness resources for Residents and Fellows. Accessed June 15, 2020. aan.com/tools-and-resources/academic-neurologists-researchers/program-and-fellowship-director-resources/residency-program-wellness/.

[26] Ramanan VK, Inbarasu JD, Jackson LM, Jones LK Jr, Klaas JP. Promoting well-being among neurology residents: a data-driven approach. Mayo Clin Proc Innov Qual Outcomes. 2020;4(5):469-474. doi: 10.1016/j.mayocpiqo.2020.06.008. 33083696 PMC7560566

[27] Poghosyan L, Aiken LH, Sloane DM. Factor structure of the Maslach burnout inventory: an analysis of data from large scale cross-sectional surveys of nurses from eight countries. Int J Nurs Stud. 2009;4651(710):894-902; Erratum in: *Int J Nurs Stud*. 2014;51(10):1416-1417. doi: 10.1016/j.ijnurstu.2009.03.004. PMC2700194 19362309

